# CRISPR-Cas9 Mediated TSPO Gene Knockout alters Respiration and Cellular Metabolism in Human Primary Microglia Cells

**DOI:** 10.3390/ijms20133359

**Published:** 2019-07-09

**Authors:** Vladimir M. Milenkovic, Dounia Slim, Stefanie Bader, Victoria Koch, Elena-Sofia Heinl, David Alvarez-Carbonell, Caroline Nothdurfter, Rainer Rupprecht, Christian H. Wetzel

**Affiliations:** 1 Molecular Neurosciences, Department of Psychiatry and Psychotherapy, University of Regensburg, 93053 Regensburg, Germany; 2 Department of Molecular Biology and Microbiology, Case Western Reserve University, Cleveland, OH 44106, USA; 3 Department of Psychiatry and Psychotherapy, University of Regensburg, 93053 Regensburg, Germany

**Keywords:** TSPO, mitochondria, knockdown, knockout, mitochondrial membrane potential, Ca^2+^ homeostasis, oxidative phosphorylation, steroid synthesis

## Abstract

The 18 kDa translocator protein (TSPO) is an evolutionary conserved cholesterol binding protein localized in the outer mitochondrial membrane. It has been implicated in the regulation of various cellular processes including oxidative stress, proliferation, apoptosis, and steroid hormone biosynthesis. Since the expression of TSPO in activated microglia is upregulated in various neuroinflammatory and neurodegenerative disorders, we set out to examine the role of TSPO in an immortalized human microglia C20 cell line. To this end, we performed a dual approach and used (i) lentiviral shRNA silencing to reduce TSPO expression, and (ii) the CRISPR/Cas9 technology to generate complete TSPO knockout microglia cell lines. Functional characterization of control and TSPO knockdown as well as knockout cells, revealed only low de novo steroidogenesis in C20 cells, which was not dependent on the level of TSPO expression or influenced by the treatment with TSPO-specific ligands. In contrast to TSPO knockdown C20 cells, which did not show altered mitochondrial function, the TSPO deficient knockout cells displayed a significantly decreased mitochondrial membrane potential and cytosolic Ca^2+^ levels, as well as reduced respiratory function. Performing the rescue experiment by lentiviral overexpression of TSPO in knockout cells, increased oxygen consumption and restored respiratory function. Our study provides further evidence for a significant role of TSPO in cellular and mitochondrial metabolism and demonstrates that different phenotypes of mitochondrial function are dependent on the level of TSPO expression.

## 1. Introduction

The translocator protein 18 kDa (TSPO) is a highly conserved multifunctional protein residing in the outer mitochondrial membrane [1]. It is expressed to varying degrees in multiple tissues, with the highest levels of expression found in steroidogenic tissues, and rather low expression in the central nervous system. In the brain, TSPO can be detected predominantly in microglia and reactive astrocytes [2], where the expression is additionally upregulated in the context of inflammation, neurodegeneration, and malignant neoplasia. In this regard, TSPO is considered as both a diagnostic biomarker, as well as a therapeutic target [3] within the promising field of theranostics [4].

Based on its initial function, the protein is named for its ability to translocate cholesterol into the mitochondrial matrix. Although the role of TSPO in de novo steroidogenesis was questioned in view of various genetic TSPO deletion models [5,6,7,8], our recent study support a role of TSPO expression as well as TSPO ligands in the regulation of pregnenolone synthesis in mouse BV-2 microglia cells [9]. Beside its suggested role on cholesterol transport and steroid synthesis [8,10,11], TSPO has been shown to be involved in the regulation of various cellular and mitochondrial functions. The multifunctional nature of TSPO also implicate mitochondrial bioenergetics (oxidative phosphorylation, OXPHOS) and metabolism [12], beta-oxidation of fatty acids [13], the production of reactive oxygen species (ROS) [14], and Ca^2+^ homeostasis [15]. Moreover, TSPO is also involved in regulating cellular downstream processes such as proliferation, survival, and apoptosis [3,16].

To investigate the functional role of TSPO within primary human microglia cells, we used immortalized C20 microglia cells as a model [17], and generated a TSPO shRNA cell line with reduced TSPO expression and two TSPO deficient knockout cell lines by means of CRIPR/Cas9 technology. By comparing C20 wildtype/scramble controls with both TSPO knockdown and TSPO deficient knockout cell lines, we analyzed the effect of TSPO expression on de novo steroidogenesis, mitochondrial respiration, mitochondrial membrane potential and Ca^2+^ homeostasis, as well as on the proliferation of the microglia cells. We found that in contrast to mouse microglia cells, TSPO has no effect on the de novo steroidogenesis in human C20 microglia cells. Moreover, TSPO strongly regulates mitochondrial bioenergetics and Ca^2+^ homeostasis as well as proliferation. Our findings are in line with the hypothesis that the particular role of this multifunctional protein in a given cell may be dependent on the level of expression as well as on the type of tissue and respective species.

## 2. Results

### 2.1. Lentiviral shRNA TSPO Knockdown and CRSPR/Cas9-Mediated Knockout of TSPO

**Figure 1:**
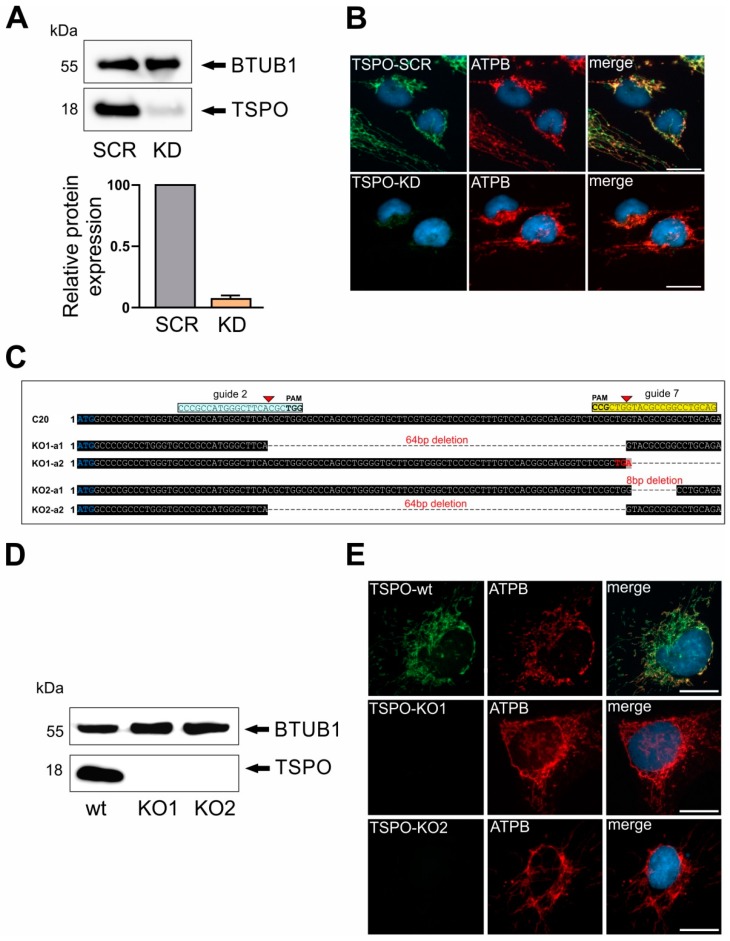
Translocator protein 18 kDa (TSPO) gene depletion and deletion in C20 human microglia cells. (**A**) TSPO gene lentiviral knockdown resulted in robust reduction of TSPO protein levels. Beta 1 tubulin was used as loading control. (**B**) Reduced levels of TSPO protein in TSPO knockdown (KD) cells in comparison to TSPO scramble (SCR) control were also evident in immunofluorescent co-staining using ATPB antibody. (**C**) Schematic diagram showing CRISPR/Cas9 guide RNA targeting sites on the exon 2 of the TSPO gene. Guide DNA targeting sites are highlighted in cyan and yellow, and start codon of TSPO gene in blue. Successful TSPO knockout in both alleles using CRISPR/Cas9 system was confirmed by DNA sequencing. (**D**) Western blot analysis of TSPO in C20 wt and KO1 and KO2 knockout cells reveal complete lack of TSPO expression. (**E**) TSPO gene deletion was further confirmed with TSPO antibody co-staining using ATPB as mitochondrial marker. Scale bars: 20 µm.

To study the role and function of TSPO in human microglia cells, we performed a lentiviral shRNA TSPO knockdown, which led to strongly reduced TSPO protein levels as demonstrated in Western blot and immunofluorescence in transduced cells (Figure 1A,B). As a mitochondrial marker, Anti-ATPB (ATP synthase subunit B) immunostaining demonstrates well the mitochondrial network in scramble control and shRNA TSPO knockdown cells. In a parallel approach, different guide RNAs (gRNAs) against the *TSPO* gene were designed (Supplementary Table S1). gRNAs were transfected into human microglial C20 cells and CRISPR/Cas9-mediated knockout of TSPO was validated by Western Blot and DNA sequencing of PCR products. In one clone, a combination of gRNA 2 and 7 deleted a 64 bp fragment, which resulted in a shift in the open reading frame in allele 1. An additional insertion of 235 bp in allele 2 generated an immediate stop codon. A second clone demonstrated an 8 bp deletion in allele 1 and a deletion of a 64 bp fragment in allele 2, which resulted in a frame shift and generated a non-functional *TSPO* gene (Figure 1C). Western blot analysis of TSPO protein expression in both clones revealed a complete loss of TSPO in the mutated cell lines KO1 and KO2, whereas C20 wildtype cells showed a prominent TSPO expression at 18 kDa (Figure 1D). TSPO knockout was also validated by immunofluorescence. Again, anti-ATPB fluorescence demonstrates nicely the mitochondrial network in C20, KO1 and KO2 cells. TSPO expression as indicated by anti-TSPO antibody is only visible in C20 wildtype, but is completely missing in KO1 and KO2 cells, validating the loss of TSPO expression in mutated C20 cells (Figure 1E). Therefore, we considered both the C20 TSPO knockdown as well as the TSPO knockout cell lines as a viable and promising tool to study the role of TSPO in human microglia cells.

### 2.2. Effect of TSPO Expression and TSPO Ligands on Steroid Synthesis in human C20 Microglia Cells

**Figure 2:**
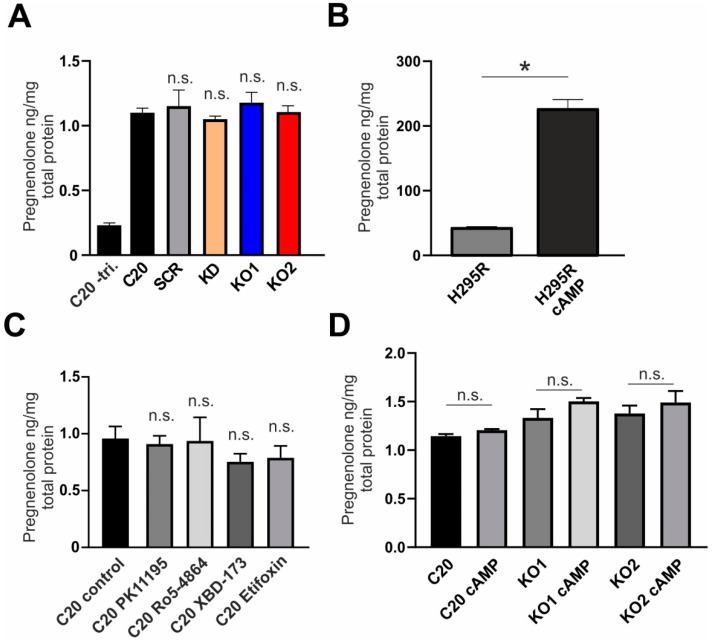
Pregnenolone synthesis in C20 and H295R cells. (**A**) There were no significant changes in basal pregnenolone production between C20 wt and TSPO knock down or knockout cells (-tri., without trilostane; n.s., no significance). (**B**) Pregnenolone synthesis in H295R steroidogenic cells, which was over 40 times higher in comparison to C20 cells under basal conditions, could be additionally upregulated in response to cAMP treatment (*p* < 0.001). (**C**) Treatment with various TSPO ligands had no effect on pregnenolone production in C20 microglia cells. (**D**) Basal pregnenolone production in C20 wt and KO cells was furthermore not affected by cAMP treatment.

Glial cells play a prominent role in neurosteroidogenesis [18,19,20]. TSPO is highly expressed in activated glia cells is reported to regulate the synthesis of pregnenolone [8,9,10,21]; thus, we aimed to investigate the role of TSPO in and the effect of TSPO ligands on pregnenolone synthesis in human microglia C20 cells. By means of pregnenolone ELISA, we detected only low levels of pregnenolone in the supernatant of unstimulated C20 wildtype cells (1.10 ± 0.1 ng/mL) after 6 h incubation with the 3β-hydroxysteroid dehydrogenase-inhibitor trilostane, which was used to prevent further metabolism of pregnenolone (Figure 2A). Interestingly, the TSPO knockdown as well as the TSPO-deficient cell lines KO1 and KO2 demonstrated a similar low pregnenolone concentration (KD: 1.05 ± 0.04 ng/mL; KO1: 1.17 ± 0.22 ng/mL; KO2: 1.10 ± 0.13 ng/mL) compared with the C20 wildtypes (Figure 2A). In contrast to C20, the steroidogenic cell line H295R (adrenocortical carcinoma cell line) [22], which we used as a positive control, released a substantially higher amount of pregnenolone leading to a concentration of 43.9 ± 0.54 ng/mL in the supernatant. Additional activation of the protein kinase A cascade by dibutyryl-cAMP markedly increased pregnenolone synthesis (Figure 2B).

Moreover, treating C20 cells with the TSPO ligands XBD173 (1 µM), Ro5-4864 (100 nM), PK11195 (100 nM) or Etifoxine (1 µM) for 24 h did not lead to increased pregnenolone concentrations (Figure 2C), indicating that the low steroid synthesizing capacity of C20 cells cannot be stimulated by TSPO ligands. Treating C20 microglia cells with 1 mM dibutyryl-cAMP for 2 h did not stimulate the pregnenolone synthesis (Figure 2D). Quantitative analysis of cytochrome P450 (CYP11A1) mRNA (the enzyme which generates pregnenolone by cleaving the side chain from cholesterol) revealed very low levels (Figure S1), supporting our observation of low steroid synthesizing capacity of our C20 cells. Together, these observations demonstrate that the human C20 microglia cell line does not produce substantial amounts of pregnenolone and indicate that TSPO and TSPO ligands do not modulate the low level of pregnenolone synthesis in C20 cells.

Thus, as a next step, we were interested in the role of TSPO in modulating mitochondrial function in human C20 cells.

### 2.3. Role of TSPO in the Modulation of the Mitochondrial Membrane Potential

As a chemo-electrical potential difference between the matrix and the intermembrane space (IMS), the mitochondrial membrane potential (MMP) serves as an indicator for the bioenergetic state of mitochondria. The MMP of human microglial C20 cells was analyzed by loading the cells with the fluorescent dye JC-1. The cationic dye accumulates in the mitochondrial membranes to an extent which is dependent on the strength of the electric field. In negatively charged (highly energized) mitochondria, JC-1 molecules form red fluorescing aggregates, while the fluorescence changes to green, when the dye molecules disaggregate into monomers in response to dissipation of the MMP. The ratio of the fluorescence signals emitted by the two states of JC-1 is then analyzed as a measure of MMP.

**Figure 3:**
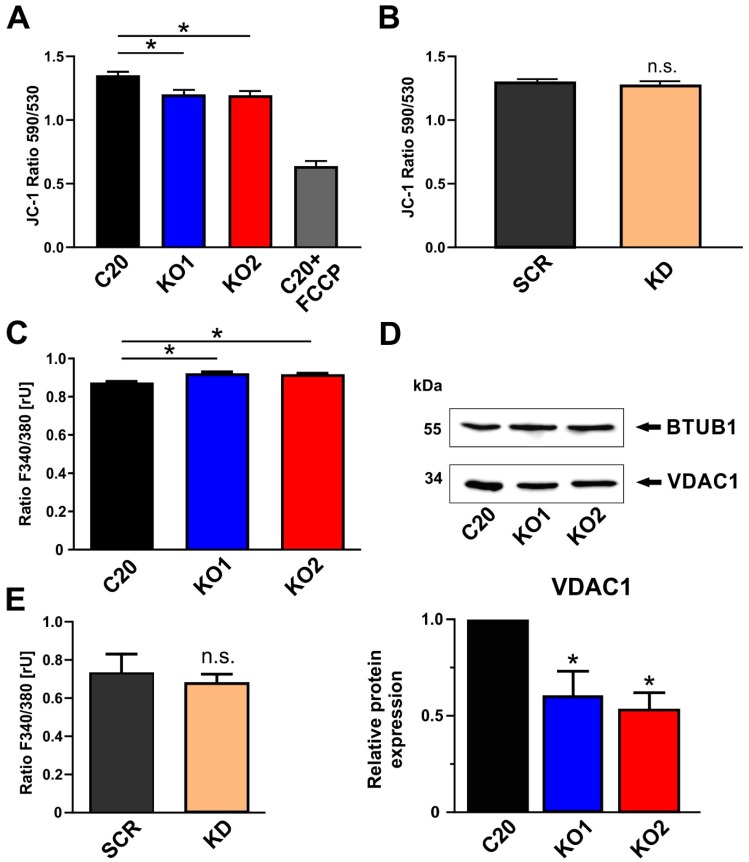
Effects of TSPO gene deletion on mitochondrial membrane potential and intracellular calcium. (**A**) TSPO gene deletion resulted in significant reduction of mitochondrial membrane potential in KO cells (*p* < 0.001) Treating the cells (C20 wildtype) with the uncoupling agent FCCP (20 µM) reduced the fluorescence ratio to about 50%, indicating the dynamic range of the JC-1 assay. (**B**) JC-1 staining was not changed after lentiviral TSPO knockdown. (**C**) TSPO knockout cells show significantly higher cytosolic Ca^2+^ levels (*p* < 0.001), suggesting an important role of TSPO in regulating Ca^2+^ homeostasis. (**D**) Deletion of TSPO gene leads to decreased expression of VDAC1 (voltage-dependent anion channel) protein in knockout cells (*p* < 0.01). (**E**) Reduction of TSPO protein level in knockdown C20 cells was not sufficient to alter the level of intracellular calcium.

We could demonstrate that KO1 and KO2 TSPO knockout cells showed a significantly reduced fluorescence of the aggregate form of JC-1 (red fluorescence) (Figure 3A). The ratio of JC-1 fluorescence (F_aggregate/monomere_) is lower in the TSPO knockout cells, thereby indicating a less hyperpolarized/negative membrane potential in the mitochondria devoid of TSPO. This significant effect could only be observed in the TSPO-deficient knockout lines. A mere reduction in TSPO expression as evident in the TSPO knockdown cell line was not sufficient to affect the MMP in our C20 microglia cells (Figure 3B).

### 2.4. Impact of TSPO on Cytosolic Ca^2+^ Homeostasis in C20 Microglia Cells

Due to their negative membrane potential, mitochondria serve as a sink for Ca^2+^ ions, contribute to the homeostasis of cytosolic Ca^2+^, and regulate intracellular Ca^2+^ signaling [23,24]. In order to investigate the effect of TSPO expression on basal cytosolic Ca^2+^ levels in C20 cells, we performed Fura-2 Ca^2+^ imaging, and found a significantly higher Fura-2 fluorescence ratio in KO1 and KO2 when compared to C20 wildtype (Figure 3C). This observation demonstrates a higher cytosolic Ca^2+^ level in TSPO knockout cells, which is in accordance with a reduced MMP as shown in Figure 3A and support a significant role of TSPO in regulating Ca^2+^-dependent mitochondrial functions. Considering a putative molecular scenario in which TSPO may function as part of a VDAC-containing supercomplex [25,26] to regulate Ca^2+^ homeostasis, we found a significant downregulation of VDAC expression in C20 TSPO deficient KO cells by performing Western blots with an anti-VDAC1 antibody (Figure 3D). However, the effect of TSPO on Ca^2+^ homeostasis was detectable only in TSPO knockout, but not knockdown C20 cells (Figure 3E).

### 2.5. Role of TSPO in Mitochondrial Respiration in Human C20 Microglial Cells

**Figure 4:**
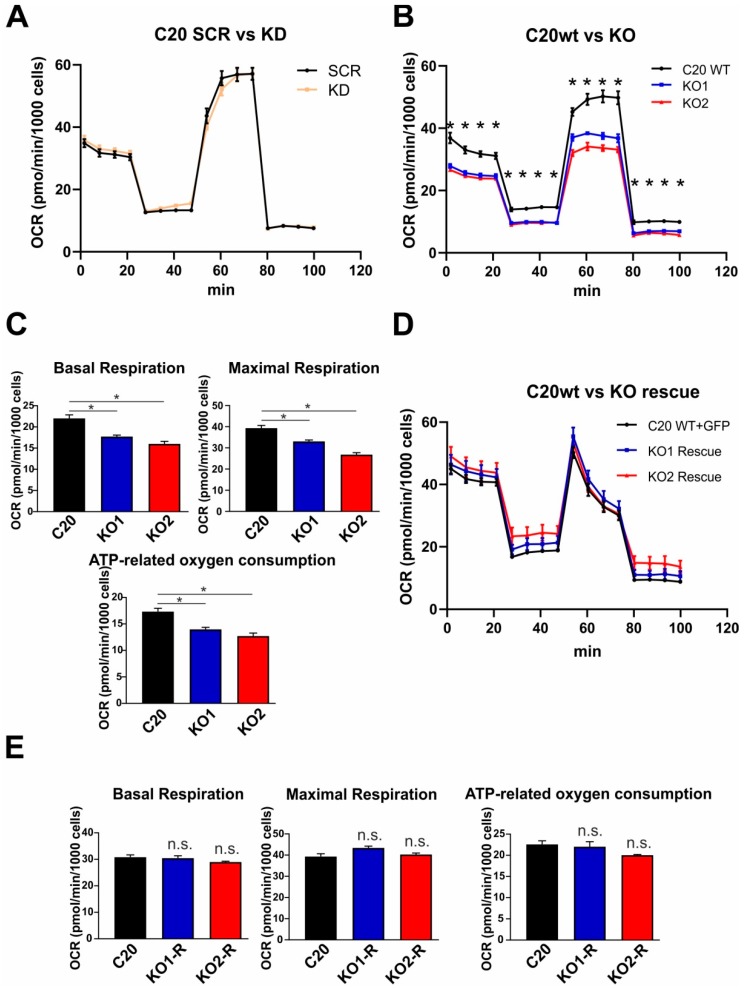
TSPO gene knockout using CRISPR/Cas9 system decreases respiration in human microglia cells. (**A**) Knockdown of TSPO did not alter respiration in C20 microglia cells (**B**) Oxygen consumption rate (OCR) was significantly reduced in several stages of respiration in TSPO KO cells (*p* < 0.001). (**C**) Basal respiration, maximal respiration, and ATP-related oxygen consumption were significantly decreased in TSPO knockout cells (*p* < 0.001). (**D**) Overexpression of TSPO in knockout cells resulted in increased respiration which did not differ from wt cells. (**E**) Basal respiration, maximal respiration, and ATP-related oxygen consumption which were reduced in KO cells, reached similar levels in TSPO overexpressing knockout cells.

The MMP and the proton gradient established at the inner mitochondrial membrane are major parameters mutually influencing the bioenergetic core functions of mitochondria, which are the electron transport and ATP synthesis at the inner membrane/matrix interface (oxidative phosphorylation, OXPHOS). Using the Seahorse XFp Flux Analyzer, we investigated the oxygen consumption in the presence of TSPO in scramble control C20 cells, and in shRNA TSPO knockdown cells. Specific substrates and selective enzyme inhibitors reveal the activity and capacity of the individual respiratory complexes and functional parameters such as the basal and maximal respiration rates, the oxygen consumption related to ATP synthesis, and the extent of respiratory coupling (oxygen consumption versus OXPHOS and ATP synthesis). However, we could not find any significant differences in the mitochondrial respiration of lentivirally transduced TSPO knockdown cells (Figure 4A). However, comparing wildtype C20 cells with the TSPO-deficient knockout cell lines KO1 and KO2, we found that TSPO expression significantly affected the mitochondrial respiration. The basal and maximal respiration rate, as well as the ATP-related oxygen consumption were significantly reduced in KO1 and KO2 TSPO knockout cell lines when compared with C20 wildtype microglia (Figure 4B,C). Moreover, rescuing the TSPO knockout by lentiviral overexpression of TSPO in the KO1 and KO2 knockout cells lines restored the TSPO protein expression in transduced cells (Figure 4D). Interestingly, KO1 and KO2 C20 cells overexpressing the TSPO protein showed an increased oxygen consumption, which was not different from the C20 (wildtype) cells after transformation with a GFP-expressing lentivirus as a control (Figure 4D). Basal and maximal respiration, as well as ATP-related oxygen consumption reached similar levels in TSPO overexpressing and GFP-expressing (wildtype) controls (Figure 4E). These findings indicate a direct effect of TSPO on mitochondrial respiration and controls for a putative CRISPR/Cas9-mediated off-target effect.

## 3. Discussion

The translocator Protein 18 kDa (TSPO) is a highly conserved outer mitochondrial membrane protein and is supposed to be involved in cholesterol transport and steroid hormone synthesis, in regulation of Ca^2+^ homeostasis and mitochondrial bioenergetics [8,15]. Species- and tissue-specific TSPO knock-out models allow to investigate the role of TSPO and the impact of TSPO deletion on steroidogenesis as well as on specific mitochondrial functions in a defined cellular context [5,6,7,8,9]. Most available data in the literature originate from rodent models. To focus on the role of TSPO in humans, we used a human microglia cell line, C20 [17], and generated two TSPO knockout lines by means of CRISP/Cas9, in addition to a lentiviral TSPO shRNA knockdown cell line. Our study demonstrated substantial expression of TSPO protein in C20 wildtype microglia cells, which is in agreement with reports describing the presence of TSPO protein in human glia cells [27,28]. Despite the significant TSPO expression in our C20 cells, we could not detect prominent levels of pregnenolone, which might be due to the low expression of cytochrome P450_scc_ expression in C20 microglia. In steroid synthesizing tissues, this enzyme is responsible for catalyzing the cleavage of cholesterol to generate pregnenolone as the first steroid hormone to be synthesized in the mitochondrial matrix. However, cytochrome P450_scc_ is reported to be nearly absent in human microglia [29], preventing substantial de novo synthesis of pregnenolone from cholesterol in these cells. In the same line of evidence, pregnenolone levels in C20 TSPO knockout cells were not different from the wildtype microglia cells, and the TSPO ligands XBD173, Ro5-4864, Pk111965, or Etifoxine did not influence pregnenolone levels neither in C20 wildtype, nor in TSPO knockout cells. However, a low basal rate of steroid synthesis in C20 cells could be demonstrated by omitting the 3β-hydroxysteroid dehydrogenase-inhibitor trilostane from the assay medium, thereby allowing further metabolism of pregnenolone. The low concentration of pregnenolone in the supernatant in the presence of trilostane was even reduced to 0.2 ng/mL when pregnenolone can be further metabolized in the absence of trilostane (Figure 2A).

Considering the abundance of TSPO in microglia cells without relevance to de novo steroid synthesis, we searched for alternative functional roles of TSPO. In this line, we found a robust effect of TSPO expression on mitochondrial respiration. The basal and the maximal respiration, as well as the ATP synthesis-related oxygen consumption were clearly and significantly reduced in TSPO deficient knockout cells compared to C20 wildtype microglia. Our current approach to compare a lentiviral shRNA knockdown with CRISPR/Cas9-mediated TSPO knockout allowed the analysis of a clear and defined TSPO deficient phenotype. In a recent study, we used mouse BV-2 TSPO knockdown cells, and found the basal, maximal, and ATP-related oxygen consumption to be unchanged between TSPO knockdown and scramble control cells. However, evidence for TSPO being a modulator of cellular energy metabolism was already reported in an early study from Vorobjev and Zorov, which pointed to an effect of diazepam as ligand of the peripheral benzodiazepine receptor (PBR) on mitochondrial respiration in pig kidney embryonal cells [30]. Later, TSPO deficiency was correlated with reduced basal oxygen consumption in primary microglia which was isolated from genetically modified TSPO knock-out mice [5]. However, the oxygen consumption rate (OCR) in mitochondria isolated from a liver-specific conditional TSPO knock-out mouse was not different from control mitochondria [31]. Similarly, mouse MA-10 Leydig cells with eliminated TSPO gene showed no differences in OCR compared to TSPO expressing control MA-10 cells [7]. In contrast, fibroblasts from global TSPO knock-out mice showed decreased OCR [32]. Overexpression of TSPO in Jurkat cells led to the upregulation of proteins involved in electron transport and energy metabolism. This gene regulation correlates with an increased mitochondrial ATP synthesis and cell proliferation [12]. These studies indicate that TSPO is able to regulate mitochondrial energy metabolism, but also suggest that the effect of TSPO on mitochondrial function may depend on cell type and species.

Feeding NADH and FADH_2_ into the respiratory complexes of the electron transport chain in the inner mitochondrial membrane (IMM), allows the pumping of protons into the intermembrane space (IMS), thereby generating a prominent electro-chemical potential difference between the matrix and IMS [33]. This mitochondrial membrane potential (MMP) is an important functional parameter which influences ATP synthesis and Ca^2+^ homeostasis. We found that knocking out TSPO in human C20 microglia cells led to a robust and significant reduction of the mitochondrial membrane potential. The reduced MMP also correlates with lower respiratory activity (see above) and with reduced Ca^2+^ retaining capacity, which would mechanistically lead to an increased basal cytosolic Ca^2+^ level [34]. A similar effect of TSPO expression on the MMP was found in mouse BV-2 microglia, where a lentiviral knockdown of TSPO led to a reduced potential difference [9], and TSPO deficient murine fibroblasts as well as Leydig cells also demonstrated a reduced MMP [35]. In a rat model of cortical contusion, TSPO expression was correlated with the loss of MMP and affected by the TSPO agonist PK11195 [36]. In addition, TSPO has also been shown to regulate dynamic Ca^2+^ signaling in various cell types and species. Both TSPO overexpression and knockdown affected ATP-induced mitochondrial Ca^2+^ transients in canine epithelial cells and correlated with the mitochondrial membrane potential difference [15].

In general, mechanisms that are engaged in regulating MMP, Ca^2+^ homeostasis, mitochondrial respiration, and bioenergetics may involve an interaction of TSPO with other proteins such as VDAC and ANT [37,38], ACBD3, and PKA [15,39]. TSPO may modulate ATP synthase via a direct interaction between the ATP synthasome complex (composed of ATP synthase, phosphate carrier, and ANT) and the PBR complex composed of TSPO, VDAC, and ANT [12,40,41]. Importantly, VDAC constitutes the principle pathway in the OMM for the transport and exchange of ions, ADP, and ATP, as well as metabolites and substrates. Its Ca^2+^ permeability has been shown to be regulated in a TSPO-dependent way by PKA-mediated phosphorylation [15]. Interestingly, we found a reduced VDAC1 expression associated with the TSPO knockout in our C20 cells. This correlation may also basically contribute to the mechanism of TSPO action on mitochondrial physiology.

In summary, our findings demonstrate that human C20 microglia cells show only low de novo synthesis of pregnenolone. Residual pregnenolone levels in C20 do not correlate with TSPO expression or treatment with canonical TSPO ligands. However, TSPO plays a significant role in the regulation of the MMP, Ca^2+^ homeostasis, and energetic metabolism. Moreover, when considering reports showing the mammalian TSPO being able to substitute for the bacterial version in negatively regulating photosynthesis genes in response to oxygen [42], it may become evident that TSPO function varies in different species and cell types, probably depending on the dynamic interaction with different proteins and the changing architecture of interacting signaling complexes. This will be in the focus of our future studies.

## 4. Materials and Methods

### 4.1. Cell Lines and Culture Conditions

Human microglia C20 cells [17] were grown in Dulbecco’s Modified Eagle’s Medium/Nutrient Mixture F-12 Ham (Sigma Aldrich, Taufkirchen, Germany), supplemented with 10% fetal calf serum (FCS), 2mM L-glutamine, 10,000 U/mL penicillin-streptomycin at 37 °C, humidified air and 5% CO_2_. Adrenocortical carcinoma NCI-H295R cells (CLS, Eppelheim, Germany) were cultured in DMDM/F12 medium containing 15mM HEPES, 6.25 µg/mL insulin, 6.25 µg/mL transferrin, 6.25 ng/mL selenium, 1.25 mg/mL BSA, 5.35 mg/mL linoleic acid, and 2.5% Nu-Serum I, supplemented with 100 U/mL penicillin and 0.1 mg/mL streptomycin, (Thermo Fischer Scientific, Darmstadt, Germany). The cells were maintained in humidified air (5 % CO_2_) at 37 °C, and medium was changed three times a week.

### 4.2. CRISPR/Cas9-Mediated TSPO Knockout

CRISPR/Cas9 genome editing experiments in C20 cells were performed with lipofection of high-fidelity (hiFi) Cas9 (Integrated DNA Technologies, Inc.) ribonucleoprotein (RNP) complex as described previously (Vakulskas et al. 2018; DOI 10.1038/s41591-018-0137-0). Briefly, guide RNAs were designed using web tool (https://zlab.bio/guide-design-resources) (access on 28.02.2017), and combined with tracrRNA (Integrated DNA Technologies, Inc., Coralville, IA, USA) in equal amounts in IDT Duplex Buffer (30 mM HEPES, pH 7.5, 100 mM Potassium Acetate) at 1 μM concentration by heating the oligos to 95 °C for 5 min and slowly cooling to room temperature. RNP complexes were formed by the addition of HiFi Cas9 enzyme, and C20 cells were transfected using RNAiMax (Thermo Fischer Scientific, Dreieich, Germany) transfection reagent. After 2 days the cells were singled out for clonal selection. Single clones were collected, and the successful knockout was confirmed by western blot using anti rabbit TSPO antibody and sequencing of genomic DNA.

### 4.3. Lentiviral TSPO Knockdown and Overexpression of TSPO in TSPO Knockout Cells

The TSPO knockdown lentiviral expression vector (pLKO-shTSPO) was constructed by replacing the original 1.9 kb stuffer in the pLKO.1 vector using AgeI/EcoRI restriction enzymes by short hairpin double-stranded oligo using the following primers:

shTSPO-F: 5’-CCGGCCACACTCAACTACTGCGTATCTCGAGATACGCAGTAGTTGAGTGTGGTTTTTG-3’

shTSPO-R: 5’-AATTCAAAAACCACACTCAACTACTGCGTATCTCGAGATACGCAGTAGTTGAGTGTGG-3’

pLKO.1-TRC cloning vector was a gift from David Root (Addgene plasmid #10878). The design of the human TSPO shRNA was taken from the RNAi Consortium (Moffat et al., 2006) (Cambridge, UK) with the clone ID (TRCN0000060433). The scramble shRNA was a gift from David Sabatini (Addgene plasmid #1864).

The lentiviral TSPO overexpression vector was constructed by replacing the GFP gene in the pLJM1-GFP plasmid (Addgene plasmid #19319) with human TSPO using hTSPO-AgeI-F, and hTSPO-EcoRI-R primers (Supplementary Table S1).

Twenty-four hours before transfection, 2.2 × 10^6^ HEK293T cells were seeded onto a 100 mm culture dish. Using a standard calcium phosphate transfection protocol, 10 μg of the pLKO.1-shTSPO or pLJM1-TSPO vector, 7.5 μg of psPAX2 (Addgene plasmid #12260), and 2.5 μg of pMD2.G (Addgene plasmid #12259) plasmid were co-transfected into HEK293T cells. Virus-containing supernatants were collected 48 h after transfection, and were immediately aliquoted and stored at −80 °C. The titer of lentiviral preparation was determined with colony formation assay using 2 µg/mL puromycin selection and 0.1% Crystal Violet staining solution. Lentiviral transduction was performed by spinoculation at 2400 rpm for 60 min by adding virus solution to cells at the multiplicity of infection of 3–5, which did not show toxic effects on the cells, in the presence of 8 µg/mL polybrene. Fresh C20 culture medium containing a 2 µg/mL of puromycin was added to cells 24 h after infection; cells remained under selection until all the mock-transfected cells died. Surviving cells were pooled and cultured for further analysis.

### 4.4. Immunofluorescence

C20 wildtype or TSPO knockdown/knockout cells were grown for 24 h on sterile glass coverslips and fixed for 10 min at room temperature with 4% (w/v) paraformaldehyde (Carl Roth GmbH, Karlsruhe, Germany). After washing, cells were permeabilized with blocking/permeabilization solution (10% (v/v) goat serum, 0.5% (v/v) Triton X-100 in 1 × PBS) for 20 min. Cells were then incubated overnight, at 4 °C with rabbit-anti-TSPO antibody (ab109497) and mouse-anti-ATPB antibody (ab14730), both from Abcam, Cambridge, UK, diluted 1:1000 in 2% goat serum and 0.1% Triton X-100 in 1 × PBS. After three additional washing steps, cells were incubated for 1 h with secondary antibodies conjugated with Alexa Fluor 488 and Cy3 (Life Technologies, Carlsbad, CA, USA, both diluted 1:1000 in 2% goat serum and 0.1% Triton X-100 in 1 × PBS. Nuclei were labeled with Hoe33342 (AppliChem, Darmstadt, Germany) at a final concentration of 0.1 ug/mL in 1 x PBS. Finally, cells were mounted with confocal matrix (Micro Tech Lab, Graz, Austria) and examined with an inverted fluorescence microscope (Observer.Z1, ZEISS, Jena, Germany). An XBO 175 W served as the light source (Lambda DG4, Sutter instruments, Novato, CA, USA). Images were taken by a ZEISS AxioCam MRm CCD camera using the ZEN software (ZEISS).

### 4.5. Western Blotting

Whole cell protein samples were sonicated and boiled in RIPA buffer and total protein was quantified using a micro-BCA colorimetric assay (Pierce, Thermo Fischer Scientific, Dreieich, Germany). Protein samples were separated by SDS-polyacrylamide gel electrophoresis on 15% gels and subsequently transferred onto Immobilon^®^-P PVDF membrane (Millipore, Bedford, MA, USA). Incubation of rabbit-anti-TSPO (ab109497), mouse-anti-ATPB (ab14730), and mouse-anti-VDAC1 (ab186321) antibodies, all from Abcam, Cambridge, UK, diluted were performed O.N. at 4 °C.

### 4.6. RNA Isolation, Reverse Transcription and Quantitative Real-time RT-PCR

Total RNA was extracted using RNA Plus Kit (Macherey-Nagel, Düren, Germany) according to the manufacturer’s instructions. First strand cDNA synthesis from 1 μg of total RNA was performed with QuantiTect Reverse Transcription Kit (Qiagen, Hilden, Germany). Quantitative RT-PCR experiments were performed with Rotor-Gene-Q machine (Qiagen, Hilden, Germany) using the 1x Takyon SYBR Master Mix (Eurogentec, Köln, Germany), and specific intron-spanning primers, listed in Supplemental Table S1. Measurements were performed in triplicate and results were analyzed with a Rotor-Gene-Q software version 2.3 (Qiagen, Hilden, Germany) applying the ΔΔCt method for relative quantification.

### 4.7. Pharmacology

The concentration of compounds in the experiments (1 µM XBD173, 100 nM PK11195, 100 nM Ro5-4864, 1 µM Etifoxine) were adjusted according to [9]. The compounds were dissolved in ethanol and diluted with assay buffer to the final working concentration (ethanol concentration below 1:1000). All treatments were compared with solvent control.

### 4.8. Pregnenolone ELISA

5 × 10^4^ C20 wildtype or TSPO knockout cells were grown in Dulbecco’s Modified Eagle’s Medium/Nutrient Mixture F-12 Ham (Sigma Aldrich, Taufkirchen, Germany), supplemented with 10% fetal calf serum (FCS), 2mM L-glutamine, 10 000U/mL penicillin-streptomycin at 37 °C, humidified air and 5% CO_2_, and incubated with XBD (1 µM), PK11195 (100 nm), Ro5-4864 (100 nm) or Etifoxine (1 µM). After 16 h, cells were washed with PBS, and kept in pregnenolone assay buffer (140 mM NaCl, 5 mM KCl, 1.8 mM CaCl_2_, 1 mM MgSO_4_, 10 mM glucose, and 10 mM HEPES) containing 25 µM trilostane (Sigma Aldrich) to inhibit further metabolism of pregnenolone. Cells were treated again with TSPO ligands for additional 6 h. Then, supernatants were analyzed using an enzyme-linked immunosorbent assay (ELISA) for pregnenolone quantification, according to the manufacturer’s recommendations (Pregnenolone ELISA, IBL International, Hamburg, Germany). Assays were read with a Tecan Spectra microplate reader (Tecan, Crailsheim, Germany) at 450 nm. Data were analyzed by Magellan Data Analysis Software (Tecan, Version 2.0) [9].

### 4.9. Mitochondrial Membrane Potential

2.5 × 10^4^ C20 wildtype or TSPO knockout cells were seeded on sterile glass coverslips (diameter 25 mm), placed in 6-well plates, and grown overnight in Dulbecco’s Modified Eagle’s Medium/Nutrient Mixture F-12 Ham at 37 °C, humidified air and 5% CO_2_. Cells were loaded with 200 nM JC-1/Pluronic (Life Technologies) in Opti-MEM (Life Technologies, Carlsbad, CA, USA) for 30 min at 37 °C, humidified air and 5% CO_2_. For imaging, coverslips were washed with assay buffer (140 mM NaCl, 5 mM KCl, 1.8 mM CaCl_2_, 1 mM MgSO_4_, 10 mM glucose, and 10 mM HEPES) and mounted in a chamber on the inverted microscope (ZEISS Observer Z.1, Jena, Germany). A Lambda DG4 high-speed wavelength switcher (Sutter instruments, Novato, USA) allowed the excitation of JC-1 480/36 nm. The emitted light was filtered at 537/42 nm and 620/60 nm for green or red fluorescence, respectively, and finally detected by a CCD camera (AxioCam MRm, ZEISS, Jena, Germany). Mitochondrial membrane potential was analyzed as ratio of red versus green fluorescence intensity in regions of interest, drawn over selected cells in the visual field using the Zen imaging software (ZEISS) and ImageJ.

### 4.10. Ca^2+^ Imaging

25 × 10^3^ C20 wildtype or TSPO knockout cells were grown in 96-well dishes and loaded with 2 µM Fura-2/AM (Life Technologies, Carlsbad, CA, USA) in opti-MEM for 45 min at 37 °C and 5% CO_2_. Cytosolic Ca^2+^ concentration in C20 cells was assessed by measuring the fluorescence at 510 nm after excitation at 340 or 380 nm in a spectrofluorometer (Tecan Spark, Männedorf, Switzerland).

### 4.11. Mitochondrial Respirometry

3 × 10^4^ C20 wildtype or TSPO knockout cells were grown in XFp 8-well miniplates (Agilent Technologies, Waldbronn, Germany) at 37 °C, humidified air and 5% CO_2_. Cartridges were prepared according to the manufacture’s recommendations. The XFp Cell Mito Stress Test Kit (Agilent Technologies, Walsbronn, Germany) contained the mitochondrial stress compounds oligomycin (1 µM), FCCP (2 µM), and rotenone/antimycin A (1 µM). Oxygen consumption rate (OCR) and extracellular acidification rate (ECAR) were measured by means of a XFp Seahorse Flux Analyzer (Agilent Technologies).

### 4.12. Statistical Analysis

Data were expressed as mean ± standard error of the mean. Statistical analysis was performed with IBM SPSS Statistics 25 and GraphPad Prism8. Statistical significance was assessed using independent samples t-test, Mann-Whitney U test, or ANOVA, combined with post hoc tests for multiple comparisons. Results were regarded statistically significant for *p* < 0.05.

## Supplementary Materials

Supplementary materials can be found at https://www.mdpi.com/1422-0067/20/13/3359/s1.

## Abbreviations

TSPO	Translocator protein 18 kDa
OXPHOS	Oxidative phosphorylation
MMP	Mitochondrial membrane potential
ROS	Reactive oxygen consumption
VDAC	Voltage-dependent anion channel
IMM	Inner mitochondrial membrane
OMM	Outer mitochondrial membrane
KD	Knockdown
KO	Knockout
