# Supplementary material for: CRISPR-Cas9 Mediated TSPO Gene Knockout alters Respiration and Cellular Metabolism in Human Primary Microglia Cells
Source: Int J Mol Sci. 2019 Jul 9;20(13):3359. doi: 10.3390/ijms20133359 (PMC6651328; doi:10.3390/ijms20133359)
Supplement: Supplementary file 1 [file ijms-20-03359-s001.zip › ijms-538121-supplementary/Supp Table 1.pdf]

Supplementary Table 1. Primers used for PCR, sequencing, cloning, CRISPR/Cas9 and qRT-PCR

Primer name	Forward primer (5´-3´)	Reverse primer (5´-3´)
Guide #2	TCTGCAGGCCGGCGTACCAGCGG	\
Guide #7	GCCCGCCATGGGCTTCACGCTGG	\
TSPO-ex2	CTGGAAATGCGTTCACTCAG	GCCTGGAGAAGACCCTCTGT
CYP11A1	TGGGTCGCCTATCACCAGTA	AAGTTCTTGGTGGCCTCTGG
TSPO	TCTTTGGTGCCCGACAAAT	GGTACCAGGCCACGGTAGT
HPRT1	TTGCTTTCCTTGGTCAGGCA	ATCCAACACTTCGTGGGGTC
hTSPO-AgeI-F	atACCGGTgcagccatggccccgcc	\
hTSPO-EcoRI-R	GAATTCtcactctggcagccgccgt	\
