# Supplementary figures and images for: CRISPR-Cas9 Mediated TSPO Gene Knockout alters Respiration and Cellular Metabolism in Human Primary Microglia Cells
Source: Int J Mol Sci. 2019 Jul 9;20(13):3359. doi: 10.3390/ijms20133359 (PMC6651328; doi:10.3390/ijms20133359)

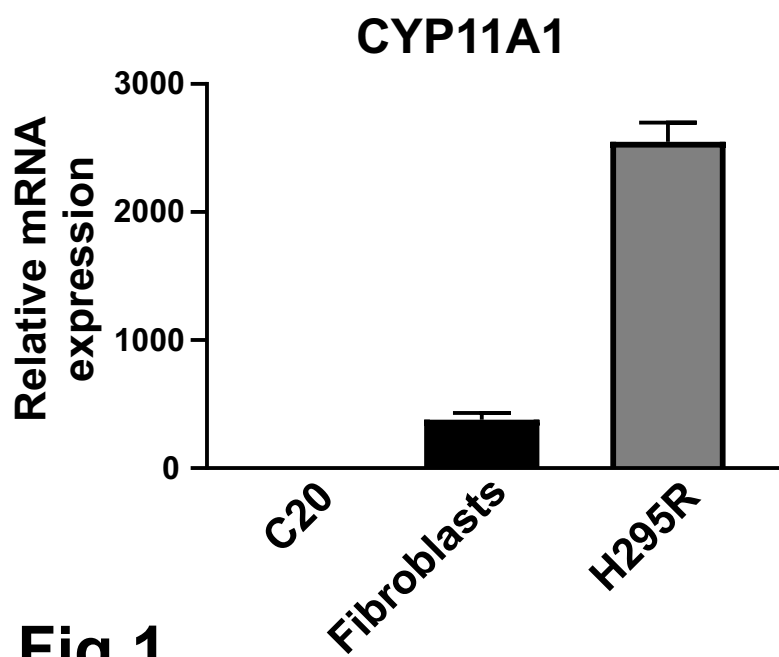

**Supp. Fig 1.**

Supplement: Supplementary file 1 [file ijms-20-03359-s001.zip › ijms-538121-supplementary/Sup Fig 1.pdf]
